# 5-(4-Meth­oxy­phen­yl)-3-(pyridin-2-yl)-4,5-dihydro-1*H*-pyrazole-1-carbothio­amide

**DOI:** 10.1107/S1600536811050033

**Published:** 2011-11-30

**Authors:** Phonpawee Nonthason, Thitipone Suwunwong, Suchada Chantrapromma, Hoong-Kun Fun

**Affiliations:** a Crystal Materials Research Unit, Department of Chemistry, Faculty of Science, Prince of Songkla University, Hat-Yai, Songkhla 90112, Thailand; b X-ray Crystallography Unit, School of Physics, Universiti Sains Malaysia, 11800 USM, Penang, Malaysia

## Abstract

In the title compound, C_16_H_16_N_4_OS, the dihedral angle between the pyridine and benzene rings is 81.08 (6)°. The pyrazole ring makes dihedral angles of 12.36 (7) and 87.96 (6)°, respectively, with the pyridine and benzene rings. In the crystal, mol­ecules are linked by N—H⋯O and N—H⋯S hydrogen bonds and a weak C—H⋯S inter­action into a layer parallel to the *ab* plane. Weak C—H⋯π and π–π inter­actions [centroid–centroid distances = 3.7043 (9) and 3.8120 (7) Å] are also observed.

## Related literature

For bond-length data, see: Allen *et al.* (1987 ▶). For a related structure, see: Fun *et al.* (2011 ▶). For background to and applications of pyrazoline derivatives, see: Amir *et al.* (2008 ▶); Bai *et al.* (2007 ▶); Gong *et al.* (2011 ▶); Husain *et al.* (2008 ▶); Ji & Shi (2006 ▶); Manna & Agrawal (2009 ▶); Shoman *et al.* (2009 ▶).
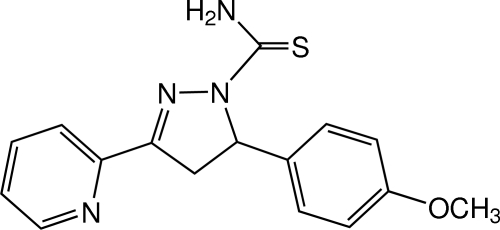


## Experimental

### 

#### Crystal data

C_16_H_16_N_4_OS
*M* *_r_* = 312.40
Triclinic, 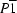

*a* = 6.2434 (2) Å
*b* = 9.9348 (4) Å
*c* = 13.6564 (6) Å
α = 107.762 (1)°
β = 99.506 (1)°
γ = 94.331 (1)°
*V* = 788.43 (5) Å^3^
*Z* = 2
Mo *K*α radiation
μ = 0.21 mm^−1^
*T* = 297 K
0.48 × 0.34 × 0.19 mm

#### Data collection

Bruker SMART APEXII CCD diffractometer
Absorption correction: multi-scan (*SADABS*; Bruker, 2009 ▶) *T*_min_ = 0.906, *T*_max_ = 0.961
19134 measured reflections
4529 independent reflections
3927 reflections with *I* > 2σ(*I*)
*R*_int_ = 0.019

#### Refinement

*R*[*F* ^2^ > 2σ(*F* ^2^)] = 0.037
*wR*(*F* ^2^) = 0.122
*S* = 1.05
4529 reflections
208 parameters
H atoms treated by a mixture of independent and constrained refinement
Δρ_max_ = 0.31 e Å^−3^
Δρ_min_ = −0.18 e Å^−3^

### 

Data collection: *APEX2* (Bruker, 2009 ▶); cell refinement: *SAINT* (Bruker, 2009 ▶); data reduction: *SAINT*; program(s) used to solve structure: *SHELXTL* (Sheldrick, 2008 ▶); program(s) used to refine structure: *SHELXTL*; molecular graphics: *SHELXTL*; software used to prepare material for publication: *SHELXTL* and *PLATON* (Spek, 2009 ▶).

## Supplementary Material

Crystal structure: contains datablock(s) global, I. DOI: 10.1107/S1600536811050033/is5010sup1.cif

Structure factors: contains datablock(s) I. DOI: 10.1107/S1600536811050033/is5010Isup2.hkl

Supplementary material file. DOI: 10.1107/S1600536811050033/is5010Isup3.cml

Additional supplementary materials:  crystallographic information; 3D view; checkCIF report

## Figures and Tables

**Table 1:** Hydrogen-bond geometry (Å, °) *Cg*3 is the centroid of the C9–C14 ring.

*D*—H⋯*A*	*D*—H	H⋯*A*	*D*⋯*A*	*D*—H⋯*A*
N4—H2*N*4⋯O1^i^	0.845 (19)	2.278 (19)	3.0238 (18)	147.4 (17)
N4—H1*N*4⋯S1^ii^	0.839 (19)	2.603 (19)	3.4090 (13)	161.6 (18)
C14—H14*A*⋯S1^iii^	0.93	2.82	3.7033 (14)	158
C1—H1*A*⋯*Cg*3^iv^	0.93	2.60	3.5005 (15)	162

Symmetry codes: (i) 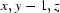; (ii) 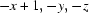; (iii) 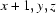; (iv) 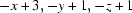.

**Fig. 1.**
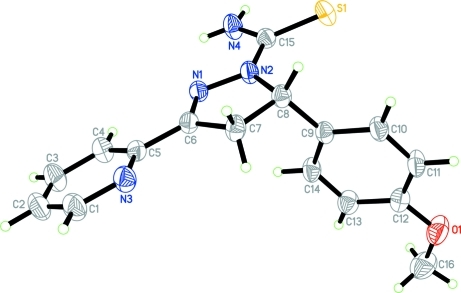
The molecular structure of the title compound, showing 50% probability displacement ellipsoids and the atom-numbering scheme.

**Fig. 2.**
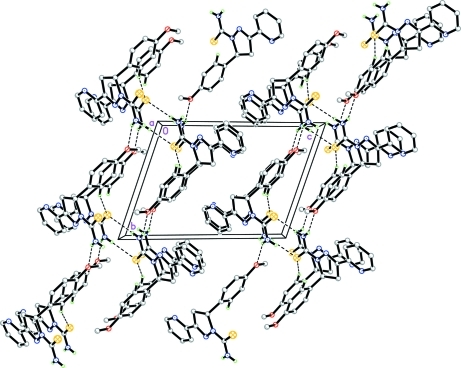
A crystal packing diagram of the title compound viewed along the a axis. For the sake of clarity, only H atoms involved in the hydrogen bonds were shown. Hydrogen bonds were drawn as dashed lines.

## supplementary crystallographic information

### Comment

Pyrazoline derivatives which contain two N atoms in their 5-membered heterocyclic structures are ultilised in bioactivity studies for their antimicrobial (Manna & Agrawal, 2009), antiamoebic (Husain *et al.*, 2008), anti-inflammatory (Amir *et al.*, 2008; Shoman *et al.*, 2009) and analgesic (Amir *et al.*, 2008) properties as well as in optical studies involving fluorescence dyes (Ji & Shi, 2006; Bai *et al.*, 2007) and fluorescent sensors (Gong *et al.*, 2011). For our research on the biological properties of pyrazoline derivatives, the title compound (I) was synthesized from the cyclization reaction of the heteroaryl chalcone derivative and thiosemicarbazide. Crystals of (I) were grown in order to study the structural and activity relationship with another pyrazoline derivative (Fun *et al.*, 2011).

In the title molecule (Fig. 1), C_16_H_16_N_4_OS, the dihedral angle between the pyridine and benzene ring is 81.08 (6)°, whereas the pyrazole ring makes dihedral angles of 12.36 (7) and 81.08 (6)° with the pyridine and benzene rings, respectively. The carbothioamide unit lies on the same plane with pyrazole ring with an *r.m.s*. of 0.0468 (1) Å for the eight non H atoms (C6, C7, C8, C15, N1, N2, N4 and S1). The methoxy group is co-planar with its attached benzene ring with a torsion angle C16–O1–C12–C13 = -0.5 (2)° and an *r.m.s*. of 0.0102 (1) Å for the eight non H atoms. Bond distances of (I) are in normal range (Allen *et al.*, 1987).

In the crystal packing (Fig. 2), the molecules are linked by N—H···O, and N—H···S hydrogen bonds as well as with weak C—H···S interactions (Table 1) into a layer parallel to the *ab* plane. π–π interactions with the distances of *Cg*1···*Cg*2 (-1 + *x*, *y*, *z*) = 3.8120 (7) Å and *Cg*2···*Cg*2 (3 - *x*, -*y*, 1 - *z*) = 3.7043 (9) Å are also present. *Cg*1 and *Cg*2 are the centroids of N1/N2/C8/C7/C6 and N3/C1–C5 rings, respectively.

### Experimental

The title compound was synthesized by the cyclization reaction of *E*-3-(4-methoxyphenyl)-1-(pyridin-2-yl)prop-2-en-1-one (0.24 g, 1 mmol) with excess thiosemicarbazide (0.18 g, 2 mmol) in a solution of KOH (0.11 g, 2 mmol) in ethanol (10 ml). The reaction mixture was vigorously stirred and refluxed for 3 h. The pale-yellow solid of the title compound obtained after cooling off the reaction was then filtered off under vacuum. Pale yellow block-shaped single crystals of the title compound suitable for X-ray structure determination were recrystallized from methanol/ethanol (1:2 *v*/*v*) by slow evaporation of the solvent at room temperature after several days (m.p. 468–469 K).

### Refinement

Amide H atoms were located in a difference maps and refined freely [N—H = 0.847 (18) and 0.84 (2) Å]. The remaining H atoms were positioned geometrically and allowed to ride on their parent atoms, with C—H = 0.93 Å for aromatic, 0.98 Å for CH, 0.97 Å for CH_2_ and 0.96 Å for CH_3_. The *U*_iso_(H) values were constrained to be 1.5*U*_eq_(C) for methyl H atoms and 1.2*U*_eq_(C) for the remaining H atoms. A rotating group model was used for the methyl groups.

### Crystal data

**Table:**

C_16_H_16_N_4_OS	*Z* = 2
*M**_r_* = 312.40	*F*(000) = 328
Triclinic, *P*1	*D*_x_ = 1.316 Mg m^−^^3^
Hall symbol: -P 1	Melting point = 468–469 K
*a* = 6.2434 (2) Å	Mo *K*α radiation, λ = 0.71073 Å
*b* = 9.9348 (4) Å	Cell parameters from 4529 reflections
*c* = 13.6564 (6) Å	θ = 1.6–30.0°
α = 107.762 (1)°	µ = 0.21 mm^−^^1^
β = 99.506 (1)°	*T* = 297 K
γ = 94.331 (1)°	Block, yellow
*V* = 788.43 (5) Å^3^	0.48 × 0.34 × 0.19 mm

### Data collection

**Table:**

Bruker SMART APEXII CCD diffractometer	4529 independent reflections
Radiation source: fine-focus sealed tube	3927 reflections with *I* > 2σ(*I*)
graphite	*R*_int_ = 0.019
Detector resolution: 8.33 pixels mm^-1^	θ_max_ = 30.0°, θ_min_ = 1.6°
ω scans	*h* = −8→8
Absorption correction: multi-scan (*SADABS*; Bruker, 2009)	*k* = −13→13
*T*_min_ = 0.906, *T*_max_ = 0.961	*l* = −19→18
19134 measured reflections	

### Refinement

**Table:**

Refinement on *F*^2^	Primary atom site location: structure-invariant direct methods
Least-squares matrix: full	Secondary atom site location: difference Fourier map
*R*[*F*^2^ > 2σ(*F*^2^)] = 0.037	Hydrogen site location: inferred from neighbouring sites
*wR*(*F*^2^) = 0.122	H atoms treated by a mixture of independent and constrained refinement
*S* = 1.05	*w* = 1/[σ^2^(*F*_o_^2^) + (0.0685*P*)^2^ + 0.1373*P*] where *P* = (*F*_o_^2^ + 2*F*_c_^2^)/3
4529 reflections	(Δ/σ)_max_ = 0.001
208 parameters	Δρ_max_ = 0.31 e Å^−^^3^
0 restraints	Δρ_min_ = −0.18 e Å^−^^3^

### Special details

**Table:**

Geometry. All esds (except the esd in the dihedral angle between two l.s. planes) are estimated using the full covariance matrix. The cell esds are taken into account individually in the estimation of esds in distances, angles and torsion angles; correlations between esds in cell parameters are only used when they are defined by crystal symmetry. An approximate (isotropic) treatment of cell esds is used for estimating esds involving l.s. planes.
Refinement. Refinement of F^2^ against ALL reflections. The weighted R-factor wR and goodness of fit S are based on F^2^, conventional R-factors R are based on F, with F set to zero for negative F^2^. The threshold expression of F^2^ > 2sigma(F^2^) is used only for calculating R-factors(gt) etc. and is not relevant to the choice of reflections for refinement. R-factors based on F^2^ are statistically about twice as large as those based on F, and R- factors based on ALL data will be even larger.

### Fractional atomic coordinates and isotropic or equivalent isotropic displacement parameters (Å^2^)

**Table:**

	*x*	*y*	*z*	*U*_iso_*/*U*_eq_	
S1	0.50444 (5)	0.19990 (4)	0.13335 (2)	0.04687 (11)	
O1	1.0431 (2)	0.76342 (12)	0.12097 (10)	0.0645 (3)	
N1	1.06459 (15)	0.13465 (10)	0.27835 (7)	0.03555 (19)	
N2	0.88621 (16)	0.19975 (9)	0.25049 (8)	0.0376 (2)	
N3	1.48004 (19)	0.27191 (11)	0.50833 (8)	0.0458 (2)	
N4	0.7587 (2)	−0.00487 (12)	0.11494 (10)	0.0497 (3)	
H2N4	0.873 (3)	−0.0389 (18)	0.1330 (13)	0.053 (4)*	
H1N4	0.667 (3)	−0.046 (2)	0.0593 (15)	0.063 (5)*	
C1	1.6684 (2)	0.24072 (15)	0.55409 (11)	0.0537 (3)	
H1A	1.7341	0.3000	0.6212	0.064*	
C2	1.7700 (2)	0.12711 (17)	0.50832 (13)	0.0568 (3)	
H2A	1.9003	0.1101	0.5437	0.068*	
C3	1.6751 (2)	0.03853 (17)	0.40878 (12)	0.0562 (3)	
H3A	1.7411	−0.0389	0.3754	0.067*	
C4	1.4800 (2)	0.06681 (14)	0.35939 (10)	0.0453 (3)	
H4A	1.4117	0.0084	0.2924	0.054*	
C5	1.38864 (18)	0.18390 (11)	0.41175 (8)	0.0354 (2)	
C6	1.18371 (18)	0.22122 (11)	0.36368 (8)	0.0347 (2)	
C7	1.0938 (2)	0.35789 (12)	0.40702 (9)	0.0416 (2)	
H7A	1.2014	0.4399	0.4191	0.050*	
H7B	1.0466	0.3630	0.4721	0.050*	
C8	0.89669 (19)	0.34932 (11)	0.31893 (9)	0.0364 (2)	
H8A	0.7627	0.3622	0.3474	0.044*	
C9	0.93345 (18)	0.45497 (11)	0.26179 (8)	0.0349 (2)	
C10	0.8047 (2)	0.56443 (13)	0.26752 (10)	0.0426 (3)	
H10A	0.6907	0.5700	0.3040	0.051*	
C11	0.8453 (2)	0.66491 (14)	0.21933 (11)	0.0486 (3)	
H11A	0.7586	0.7379	0.2239	0.058*	
C12	1.0148 (2)	0.65770 (13)	0.16395 (10)	0.0439 (3)	
C13	1.1430 (2)	0.54861 (14)	0.15695 (11)	0.0468 (3)	
H13A	1.2560	0.5423	0.1197	0.056*	
C14	1.1009 (2)	0.44869 (13)	0.20617 (10)	0.0434 (3)	
H14A	1.1875	0.3757	0.2016	0.052*	
C15	0.72789 (18)	0.12674 (12)	0.16632 (9)	0.0360 (2)	
C16	1.2161 (3)	0.7646 (2)	0.06553 (15)	0.0706 (5)	
H16A	1.2216	0.8478	0.0437	0.106*	
H16B	1.1910	0.6806	0.0050	0.106*	
H16C	1.3526	0.7662	0.1104	0.106*	

### Atomic displacement parameters (Å^2^)

**Table:**

	*U*^11^	*U*^22^	*U*^33^	*U*^12^	*U*^13^	*U*^23^
S1	0.03741 (17)	0.05216 (19)	0.04509 (18)	0.01644 (13)	0.00125 (12)	0.00828 (13)
O1	0.0804 (8)	0.0561 (6)	0.0773 (7)	0.0238 (5)	0.0297 (6)	0.0404 (6)
N1	0.0342 (4)	0.0324 (4)	0.0385 (4)	0.0070 (3)	0.0025 (3)	0.0110 (3)
N2	0.0369 (4)	0.0315 (4)	0.0401 (5)	0.0093 (3)	0.0002 (4)	0.0083 (4)
N3	0.0493 (6)	0.0392 (5)	0.0412 (5)	0.0060 (4)	−0.0046 (4)	0.0090 (4)
N4	0.0486 (6)	0.0380 (5)	0.0484 (6)	0.0120 (4)	−0.0096 (5)	0.0021 (4)
C1	0.0516 (7)	0.0504 (7)	0.0480 (7)	0.0033 (6)	−0.0122 (6)	0.0121 (5)
C2	0.0424 (7)	0.0624 (8)	0.0618 (8)	0.0117 (6)	−0.0060 (6)	0.0222 (7)
C3	0.0487 (7)	0.0586 (8)	0.0590 (8)	0.0218 (6)	0.0059 (6)	0.0147 (6)
C4	0.0450 (6)	0.0469 (6)	0.0403 (6)	0.0131 (5)	0.0037 (5)	0.0095 (5)
C5	0.0356 (5)	0.0350 (5)	0.0356 (5)	0.0037 (4)	0.0030 (4)	0.0141 (4)
C6	0.0372 (5)	0.0325 (5)	0.0344 (5)	0.0062 (4)	0.0042 (4)	0.0119 (4)
C7	0.0509 (6)	0.0340 (5)	0.0358 (5)	0.0108 (4)	0.0008 (4)	0.0084 (4)
C8	0.0388 (5)	0.0323 (5)	0.0359 (5)	0.0096 (4)	0.0054 (4)	0.0076 (4)
C9	0.0363 (5)	0.0314 (5)	0.0345 (5)	0.0102 (4)	0.0039 (4)	0.0073 (4)
C10	0.0395 (6)	0.0425 (6)	0.0495 (6)	0.0170 (5)	0.0121 (5)	0.0160 (5)
C11	0.0509 (7)	0.0433 (6)	0.0590 (7)	0.0242 (5)	0.0132 (6)	0.0218 (6)
C12	0.0509 (7)	0.0394 (6)	0.0432 (6)	0.0109 (5)	0.0073 (5)	0.0157 (5)
C13	0.0510 (7)	0.0456 (6)	0.0492 (6)	0.0157 (5)	0.0188 (5)	0.0162 (5)
C14	0.0466 (6)	0.0399 (5)	0.0492 (6)	0.0204 (5)	0.0157 (5)	0.0157 (5)
C15	0.0347 (5)	0.0363 (5)	0.0363 (5)	0.0059 (4)	0.0045 (4)	0.0118 (4)
C16	0.0854 (12)	0.0663 (10)	0.0737 (11)	0.0083 (9)	0.0283 (9)	0.0361 (9)

### Geometric parameters (Å, °)

**Table:**

S1—C15	1.6801 (11)	C5—C6	1.4657 (15)
O1—C12	1.3644 (16)	C6—C7	1.4978 (15)
O1—C16	1.419 (2)	C7—C8	1.5498 (16)
N1—C6	1.2871 (14)	C7—H7A	0.9700
N1—N2	1.3864 (12)	C7—H7B	0.9700
N2—C15	1.3536 (14)	C8—C9	1.5118 (15)
N2—C8	1.4844 (14)	C8—H8A	0.9800
N3—C1	1.3414 (17)	C9—C14	1.3846 (17)
N3—C5	1.3425 (14)	C9—C10	1.3911 (15)
N4—C15	1.3273 (15)	C10—C11	1.3825 (18)
N4—H2N4	0.847 (18)	C10—H10A	0.9300
N4—H1N4	0.84 (2)	C11—C12	1.3927 (19)
C1—C2	1.370 (2)	C11—H11A	0.9300
C1—H1A	0.9300	C12—C13	1.3848 (17)
C2—C3	1.378 (2)	C13—C14	1.3901 (18)
C2—H2A	0.9300	C13—H13A	0.9300
C3—C4	1.3834 (18)	C14—H14A	0.9300
C3—H3A	0.9300	C16—H16A	0.9600
C4—C5	1.3847 (16)	C16—H16B	0.9600
C4—H4A	0.9300	C16—H16C	0.9600
			
C12—O1—C16	118.84 (12)	N2—C8—C9	112.03 (9)
C6—N1—N2	107.90 (9)	N2—C8—C7	100.52 (8)
C15—N2—N1	119.54 (9)	C9—C8—C7	113.07 (9)
C15—N2—C8	127.01 (9)	N2—C8—H8A	110.3
N1—N2—C8	113.43 (8)	C9—C8—H8A	110.3
C1—N3—C5	116.48 (12)	C7—C8—H8A	110.3
C15—N4—H2N4	121.2 (11)	C14—C9—C10	118.46 (11)
C15—N4—H1N4	115.2 (13)	C14—C9—C8	120.98 (10)
H2N4—N4—H1N4	123.0 (17)	C10—C9—C8	120.52 (10)
N3—C1—C2	124.18 (13)	C11—C10—C9	120.44 (11)
N3—C1—H1A	117.9	C11—C10—H10A	119.8
C2—C1—H1A	117.9	C9—C10—H10A	119.8
C1—C2—C3	118.64 (12)	C10—C11—C12	120.53 (11)
C1—C2—H2A	120.7	C10—C11—H11A	119.7
C3—C2—H2A	120.7	C12—C11—H11A	119.7
C2—C3—C4	118.81 (13)	O1—C12—C13	124.58 (12)
C2—C3—H3A	120.6	O1—C12—C11	115.81 (11)
C4—C3—H3A	120.6	C13—C12—C11	119.60 (11)
C3—C4—C5	118.60 (12)	C12—C13—C14	119.22 (12)
C3—C4—H4A	120.7	C12—C13—H13A	120.4
C5—C4—H4A	120.7	C14—C13—H13A	120.4
N3—C5—C4	123.29 (11)	C9—C14—C13	121.75 (11)
N3—C5—C6	115.16 (10)	C9—C14—H14A	119.1
C4—C5—C6	121.54 (10)	C13—C14—H14A	119.1
N1—C6—C5	120.88 (10)	N4—C15—N2	115.95 (10)
N1—C6—C7	114.44 (10)	N4—C15—S1	123.24 (9)
C5—C6—C7	124.68 (10)	N2—C15—S1	120.78 (8)
C6—C7—C8	102.65 (9)	O1—C16—H16A	109.5
C6—C7—H7A	111.2	O1—C16—H16B	109.5
C8—C7—H7A	111.2	H16A—C16—H16B	109.5
C6—C7—H7B	111.2	O1—C16—H16C	109.5
C8—C7—H7B	111.2	H16A—C16—H16C	109.5
H7A—C7—H7B	109.2	H16B—C16—H16C	109.5
			
C6—N1—N2—C15	−175.98 (10)	C6—C7—C8—N2	9.45 (11)
C6—N1—N2—C8	5.41 (13)	C6—C7—C8—C9	−110.14 (10)
C5—N3—C1—C2	0.3 (2)	N2—C8—C9—C14	−50.50 (14)
N3—C1—C2—C3	0.3 (2)	C7—C8—C9—C14	62.24 (14)
C1—C2—C3—C4	−0.7 (2)	N2—C8—C9—C10	132.10 (11)
C2—C3—C4—C5	0.5 (2)	C7—C8—C9—C10	−115.16 (12)
C1—N3—C5—C4	−0.49 (19)	C14—C9—C10—C11	−0.53 (19)
C1—N3—C5—C6	−179.71 (11)	C8—C9—C10—C11	176.93 (11)
C3—C4—C5—N3	0.1 (2)	C9—C10—C11—C12	0.3 (2)
C3—C4—C5—C6	179.25 (12)	C16—O1—C12—C13	−0.5 (2)
N2—N1—C6—C5	−178.44 (9)	C16—O1—C12—C11	178.55 (14)
N2—N1—C6—C7	1.85 (13)	C10—C11—C12—O1	−178.90 (13)
N3—C5—C6—N1	−169.93 (11)	C10—C11—C12—C13	0.2 (2)
C4—C5—C6—N1	10.83 (17)	O1—C12—C13—C14	178.56 (13)
N3—C5—C6—C7	9.75 (16)	C11—C12—C13—C14	−0.5 (2)
C4—C5—C6—C7	−169.49 (11)	C10—C9—C14—C13	0.26 (19)
N1—C6—C7—C8	−7.69 (13)	C8—C9—C14—C13	−177.19 (11)
C5—C6—C7—C8	172.61 (10)	C12—C13—C14—C9	0.2 (2)
C15—N2—C8—C9	−67.76 (15)	N1—N2—C15—N4	−0.80 (16)
N1—N2—C8—C9	110.73 (10)	C8—N2—C15—N4	177.61 (11)
C15—N2—C8—C7	171.91 (11)	N1—N2—C15—S1	177.43 (8)
N1—N2—C8—C7	−9.61 (12)	C8—N2—C15—S1	−4.16 (17)

### Hydrogen-bond geometry (Å, °)

**Table:**

*Cg*3 is the centroid of the C9–C14 ring.

**Table:**

*D*—H···*A*	*D*—H	H···*A*	*D*···*A*	*D*—H···*A*
N4—H2N4···O1^i^	0.845 (19)	2.278 (19)	3.0238 (18)	147.4 (17)
N4—H1N4···S1^ii^	0.839 (19)	2.603 (19)	3.4090 (13)	161.6 (18)
C14—H14A···S1^iii^	0.93	2.82	3.7033 (14)	158
C1—H1A···Cg3^iv^	0.93	2.60	3.5005 (15)	162

Symmetry codes: (i) *x*, *y*−1, *z*; (ii) −*x*+1, −*y*, −*z*; (iii) *x*+1, *y*, *z*; (iv) −*x*+3, −*y*+1, −*z*+1.
